# Supplementary material for: CRISPR/Cas9-based editing of a sensitive transcriptional regulatory element to achieve cell type-specific knockdown of the NEMO scaffold protein
Source: PLoS One. 2019 Sep 25;14(9):e0222588. doi: 10.1371/journal.pone.0222588 (PMC6760803; doi:10.1371/journal.pone.0222588)
Supplement: S1 File — Tables A-D and Figs A-D. (DOCX) [file pone.0222588.s001.docx]

Supporting Information for

**CRISPR/Cas9-based editing of a sensitive transcriptional regulatory element to achieve cell type-specific knockdown of the NEMO scaffold protein**

Milad Babaei^1¶^ , Yuekun Liu^1¶^, Shelly M. Wuerzberger-Davis^2^, Ethan Z. McCaslin^1^, Christopher J. DiRusso^1^, Alan T. Yeo^1^, Larisa Kagermazova^1^, Shigeki Miyamoto^2^, Thomas D. Gilmore^1*^

^1^ Department of Biology, Boston University, Boston, Massachusetts, United States of America

^2^ Department of Oncology, McArdle Laboratory for Cancer Research, University of Wisconsin Carbone Cancer Center, University of Wisconsin, Madison, Wisconsin, United States of America

**Table A. Genomic target site sequence reads of dCas9 clones d2 and d3.**

| **293T cell clone** | **Total *NEMO* sequence reads** | **Total WT *NEMO* reads (%)** |
| --- | --- | --- |
| d2 | 47,208 | 47,208 (100%) |
| d3 | 24,337 | 24,337 (100%) |

**Table B. Plasmids used in this study.**

| **Plasmid Name** | **Construction or Source** |
| --- | --- |
| LentiCRISPR v2.0 | [ref. S1] and Addgene (#52961) |
| Lenti-CRISPR-gRNA1 | gRNA1 oligos were annealed and subcloned into the BsmBI site of Lenti-CRISPR v2.0 |
| Lenti-CRISPR-gRNA-C | gRNA-C oligos were annealed and subcloned into the BsmBI site of Lenti-CRISPR v2.0 |
| pCMV-dR8.91 | Gag-pol helper virus plasmid [ref. S2] |
| pCMV-VSV-G | Env helper virus plasmid (Addgene #8454) |
| Lenti-CRISPR-dCas9 | Mutations at codons 10 and 840 of Cas9 were generated by three sequential rounds of overlap PCR using the 8 Cas9-codon primers listed in Table S2. An XbaI-BamHI fragment was then used to replace the analogous wild-type Cas9 fragment in LentiCRISPRv2.0. Plasmid was deposited with Addgene (#112233), and verified by complete DNA sequencing |
| pSL1180 | Intermediate vector (Amersham) used for subcloning of dCas9 mutant fragments, prior to subcloning back into LentiCRISPRv2.0 |
| pcDNA-FLAG | [ref. S3] |
| pcDNA-FLAG-NEMO | [ref. S3] |
| pcDNA-FLAG-9-SG | [ref. S4] |
| pcDNA-FLAG-NEMO point mutations | Mutations of NEMO (D113N, R123W, L153R, R173G, R17P, and Q183H) were generated by overlap PCR using mutant NEMO primers (see Table S3). EcoRI and BamHI sites were included at the ends in the final PCR product, and products were subcloned into pcDNA-FLAG digested with EcoRI and BamHI. Mutations were verified by DNA sequencing. |

**Table C. Primers used in this study.**

| **Primer Name** | **Primer Use** | **Primer Sequence (5’-.3’)** |
| --- | --- | --- |
| gRNA-1 (pair) | CRISPR targeting of NEMO | CACCGGGAAGGGCGACCGCGAAACT  CCCTTCCCGCTGGCGCTTTGACAAA |
| gRNA-C (pair) | CRISPR targeting control | CACCGAGAGTCCGTCAGACGTGAGA  CTCTCAGGCAGTCTGCACTCTCAAA |
| NEMO Exon 2 Fwd | qPCR | GCACCTGCCTTCAGAACAGG |
| NEMO Exon 3 Rev | qPCR | ATCTGGTTGCTCTGCCGG |
| NEMO Exon 6 Fwd | qPCR | GTGAGCGGAAGCGAGGAATG |
| NEMO Exon 7 Rev | qPCR | AACGGTCTCCATCACAATCT |
| NEMO Exon 1B Fwd | RT-PCR | CGGATCCCACAGCTATGACACCGGAAG |
| NEMO Exon 2 Rev | qPCR | GAGGTGCCTATTCATCCAA |
| GAPDH Fwd (exon 7) | RT-PCR, qPCR | TGCACCACCAACTGCTTAGC |
| GAPDH Rev (exon 8) | RT-PCR, qPCR | GGCATGGACTGTGGTCATGAG |
| NEMO gExon 1B Fwd | Genomic amplification | GCCTCACACTTCTCGCCGGCTTCCC |
| NEMO gIntron 1 Rev | Genomic amplification | AGGACCACACCTGTCAGCAGAGTCCGTC |
| gDNA Fwd | Genomic amplification | CGGCGTGCTTATCATTACCGA |
| gDNA Rev | Genomic amplification | GTAGAAACAGTCCAGCTCGC |
| Cas9-Codon 10-Upstream | Cloning of Cas9 codon 10 mutation | GTTCCTGTTCCATTAACTGCG |
| Cas9-Codon 10-Fwd | Cas9 codon 10 mutation | TCGGCCTGGCCATCGGCACCAA |
| Cas9-codon 10-Rev | Cas9 codon 10 mutation | GTGCCGATGCCCAGGCCGAT |
| Cas9-Codon 10-Downstream | Cloning of Cas9 codon 10 mutation | GGGTCAGCACGATATCTTCCA |
| Cas9-codon 840-Upstream | Cloning of Cas9 codon 840 mutation | TGGAAGATATCGTGCTGACCC |
| Cas9-codon 840-Fwd | Cas9 codon 840 mutation | ACGATGTGGACGCCATCGTGCCTCAGAGC |
| Cas9-codon 840-Rev | Cas9 codon 840 mutation | GCTCTGAGGCACGATGGCGTCCACATCGT |
| Cas9-codon 840-Downstream | Cloning of Cas9 codon 840 mutation | AAGTTTGTTGCGCCGGAT |
| EcoRI-NEMO-aa2-Fwd | Subcloning of NEMO point mutations into pcDNA-FLAG | GATAGAATTCAAATAGGCACCTC  TGGAAGAGC |
| NEMO-aa419-BamHI-Rev | Subcloning of NEMO point mutations into pcDNA-FLAG | AATGGATCCCTACTCAATGCACTC  CATGACATG |
| NEMO-D113N-Fwd | Creation of mutation | GGCCTGGAGAAGCTCAATCTGAAG  AGGCAGAAG |
| NEMO-D113N-Rev | Creation of mutation | CTTCTGCCTCTTCAGATTGAGCTT  CTCCAGGCC |
| NEMO-R123W-Fwd | Creation of mutation | GCTCTGTGGGAGGTGGAGCAC |
| NEMO-R123W-Rev | Creation of mutation | CTCCACCTCCCACAGAGCCTG |
| NEMO-L153R-Fwd | Creation of mutation | TTGCTCGGGGAGCGGCAGGAGAGCCAG |
| NEMO-L153R-Rev | Creation of mutation | CTGGCTCTCCTGCCGCTCCCCGAGCAA |
| NEMO-R173G-Fwd | Creation of mutation | CAGGCTCTGGAGGGT  GGGGCCCGG |
| NEMO-R173G-Rev | Creation of mutation | CCGGGCCCCACCCT  CCAGAGCCTG |
| NEMO-R175P-Fwd | Creation of mutation | GAGGGTCGGGCTCCGG  CGGCCAGCG |
| NEMO-R175P-Rev | Creation of mutation | CGCTGGCCGCCGGAGC  CCGACCCTC |
| NEMO-Q183H-Fwd | Creation of mutation | GCAGGCGCGGCACCTG  GAGAGTGAG |
| NEMO-Q183H-Rev | Creation of mutation | CTCACTCTCCAGGTG  CCGCGCCTGC |

For NEMO mutants: the underlined bases indicate the changed codons; bases in red are the mutant bases; bases in green at the wild-type bases.

**Table D. X chromosome copy number of 293T cells.**

| **293T cell type** | **Number of X chromosomes*** |
| --- | --- |
| Parental | X (1.0%); XX (6.5%); XXX (92.5%) |
| Clone 1.1 | X (0.5%); XX (42.5%); XXX (57%) |

*Based on FISH X-chromosome analysis of 200 cells. Analysis

was performed by the WiCell Research Institute (Madsion, WI).

**Figure A**


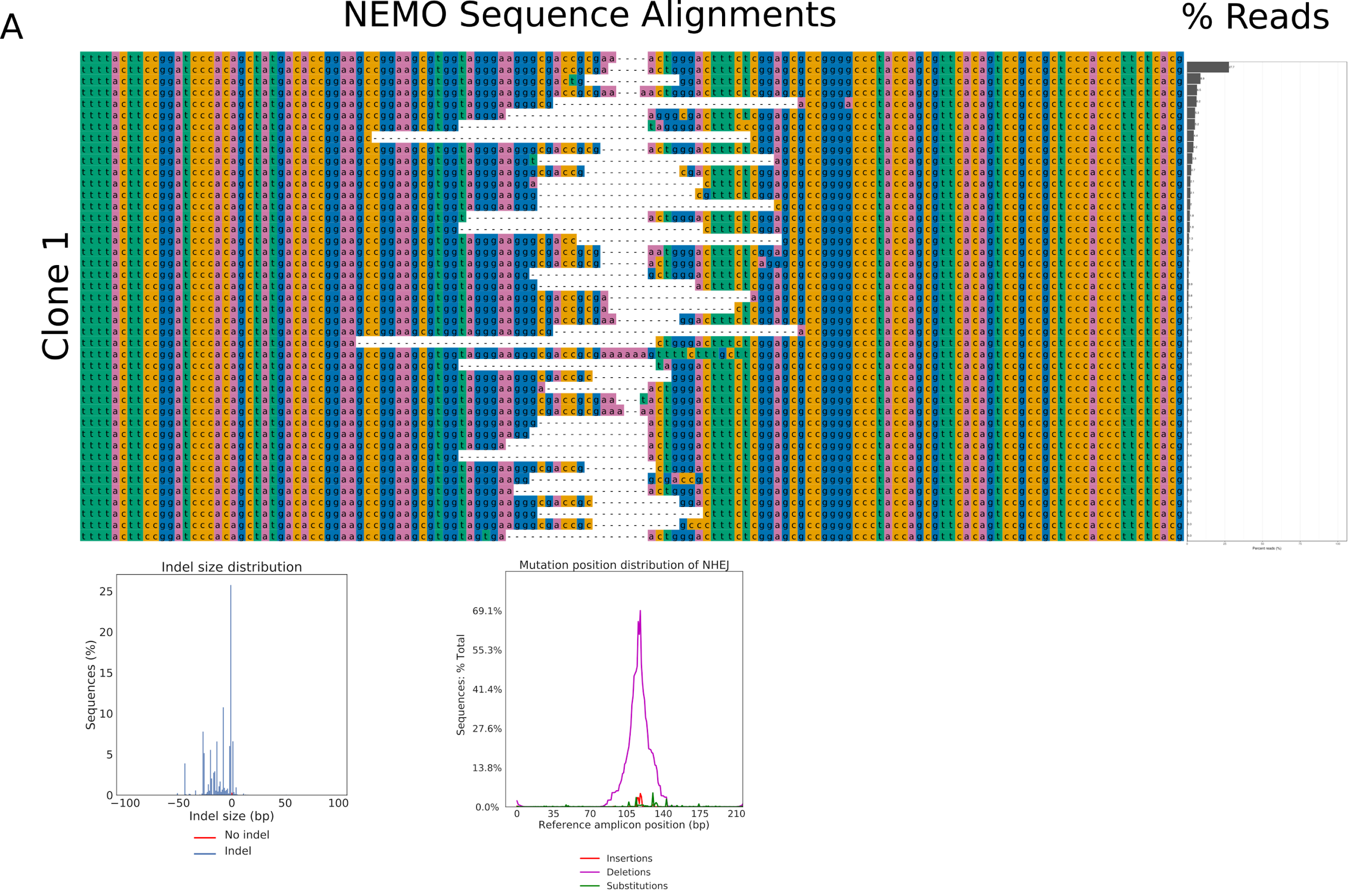


**Fig A. Full genomic profile of the targeted locus in clone 1 cells.** Sequencing of the targeted genomic locus in clone 1 cells was done by PCR amplification of the targeted site and Next generation sequencing of the PCR product. Shown are the wild-type reference sequence (top) and the 42 most abundant genomic sequences, based on 34,230 sequence reads. The percentage of reads that correspond to each sequence is shown to the right.


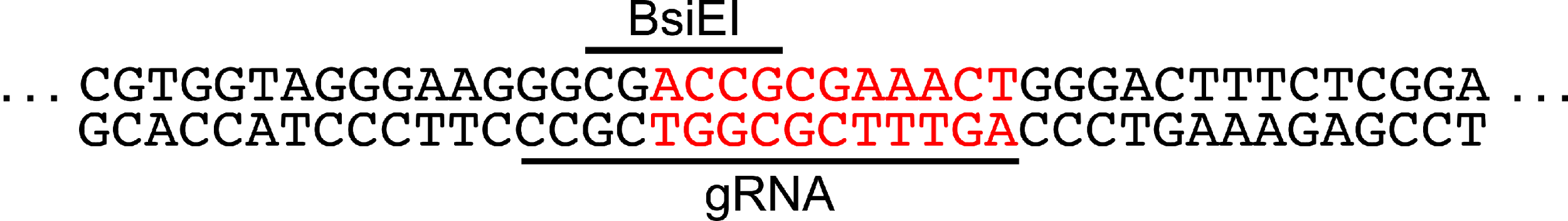
**Figure B**

**Fig B. BsiEI site (CGRYCG) in the targeted *NEMO* exon 1B core sequence (red), which was targeted by the gRNA (underlined).**

**Figure C**


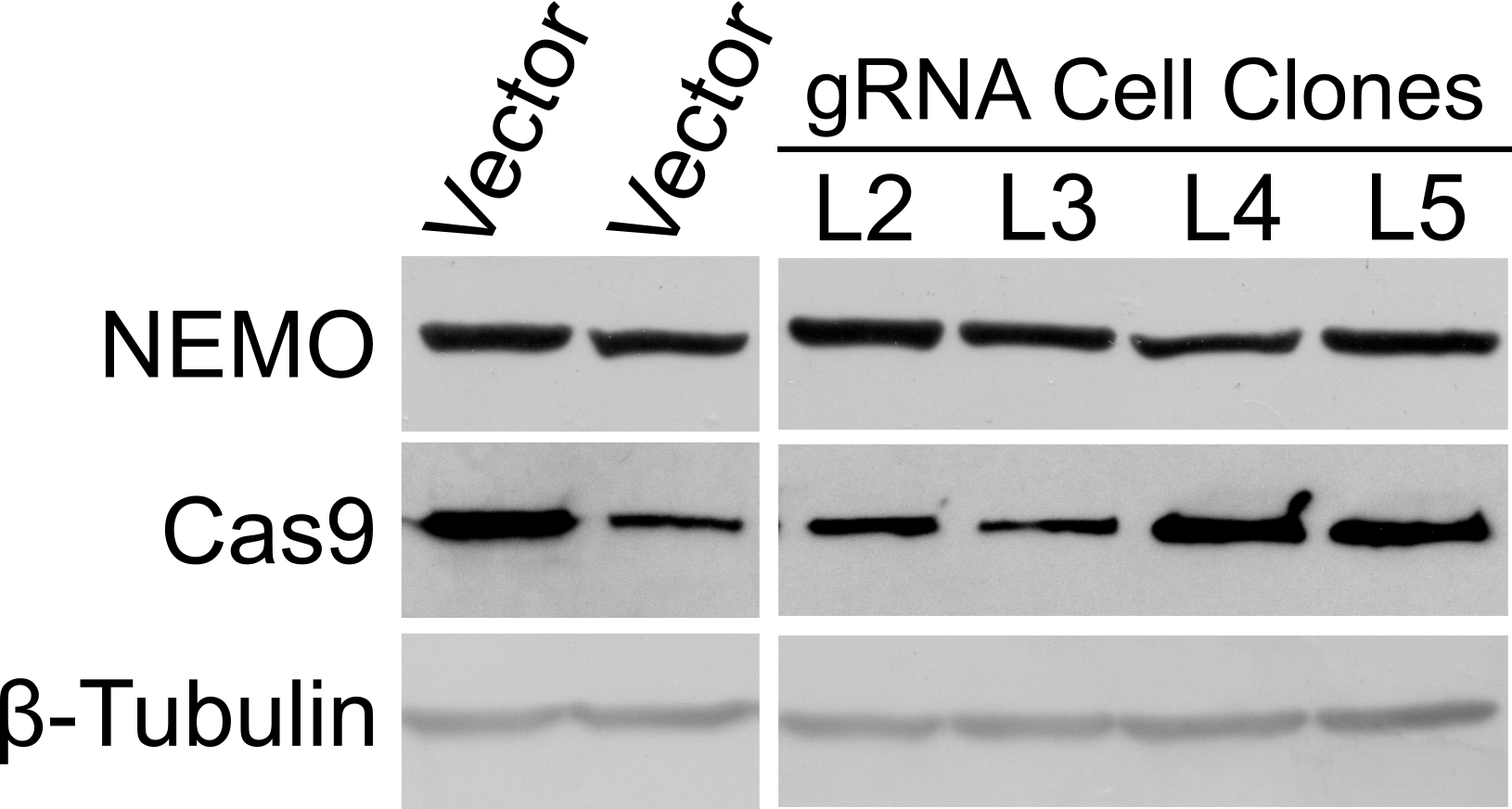


**Fig C. Targeting of the *NEMO* exon 1B promoter element does not affect NEMO expression in multiple clones of SNU-423 human liver cells.** As in Fig 5, SNU-423 cells were transfected with a LentiCRISPR2.0-Cas9 construct containing no gRNA (Vector) or the exon 1B gRNA, and transfected cells were selected with puromycin. Individual cell clones were picked and expanded. Western blotting for NEMO, Cas9, and β-tubulin was then performed on extracts from the indicated SNU-423 cell clones.

**Figure D**

**
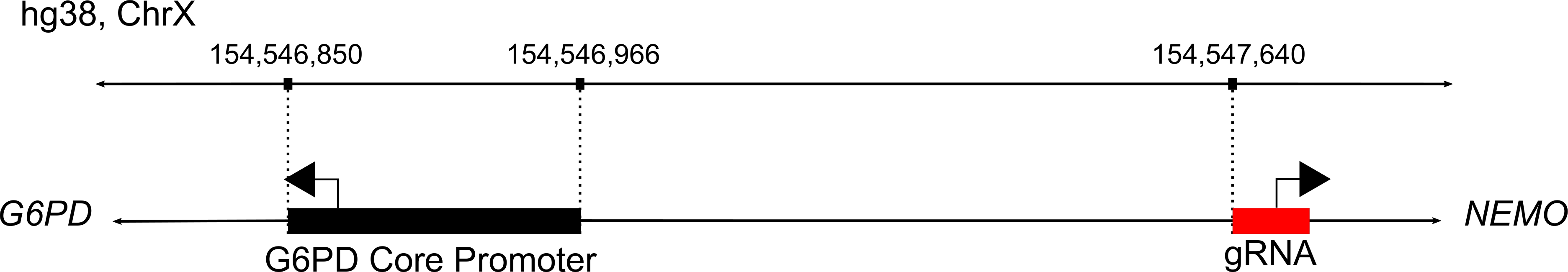
**

**Fig D. The *NEMO* promoter targeting site is located approximately 675 bp away from the start of the core promoter elements for *G6PD*.** The figure is based on Franzè et al. [ref. S5], who mapped the core promoter as consisting of three Sp family TF binding sites, a TATA box, and an initiator site near the start of transcription. The relative position of our *NEMO* exon 1B gRNA targeting element (red) is also shown. Genome numbers are based on nucleotide mapping of the X chromosome.

**Supplemental References**

S1. Sanjana NE, Shalem O, Zhang F. Improved vectors and genome-wide libraries for CRISPR screening. Nat Methods. 2014;11:783-4.

S2. Zufferey R, Nagy D, Mandel RJ, Naldini L, Trono D. Multiply attenuated lentiviral vector achieves efficient gene delivery in vivo. Nat Biotechnol. 1997; 15:871-5.

S3. Herscovitch M, Comb W, Ennis T, Coleman K, Yong S, Armstead B, et al. Intermolecular disulfide bond formation in the NEMO dimer requires Cys54 and Cys347. Biochem Biophys Res Commun. 2009;367:103-8.

S4. Shaffer R, DeMaria AM, Kagermazova L, Liu Y, Babaei M, Caban-Penix S, et al. A central conserved region of NEMO is required for IKKβ-induced conformational change and signal propagation. Biochemistry 2019;58:2906-2920.

S5. Franzè A, Ferrante MI, Fusco F, Santoro E, Martini G, Ursini MV. Molecular anatomy of the human glucose 6-phosphate dehydrogenase promoter. FEBS Lett. 1998;437:313-8.
